# The Evolution of Generosity in the Ultimatum Game

**DOI:** 10.1038/srep34102

**Published:** 2016-09-28

**Authors:** Arend Hintze, Ralph Hertwig

**Affiliations:** 1 BEACON Center for the Study of Evolution in Action, Biomedical and Physical Sciences Building, 567 Wilson Road Room 1441, East Lansing, MI 48824, USA; 2 Department of Integrative Biology, Michigan State University, 288 Farm Lane RM 203, East Lansing, MI 48824, USA; 3 Department of Computer Science and Engineering, Michigan State University, 428 South Shaw Lane RM 3115, MI 48824, USA; 4 Center for Adaptive Rationality, Max Planck Institute for Human Development, Berlin, Germany

## Abstract

When humans fail to make optimal decisions in strategic games and economic gambles, researchers typically try to explain why that behaviour is biased. To this end, they search for mechanisms that cause human behaviour to deviate from what seems to be the rational optimum. But perhaps human behaviour is not biased; perhaps research assumptions about the optimality of strategies are incomplete. In the one-shot anonymous symmetric ultimatum game (UG), humans fail to play optimally as defined by the Nash equilibrium. However, the distinction between kin and non-kin—with kin detection being a key evolutionary adaption—is often neglected when deriving the “optimal” strategy. We computationally evolved strategies in the UG that were equipped with an evolvable probability to discern kin from non-kin. When an opponent was not kin, agents evolved strategies that were similar to those used by humans. We therefore conclude that the strategy humans play is not irrational. The deviation between behaviour and the Nash equilibrium may rather be attributable to key evolutionary adaptations, such as kin detection. Our findings further suggest that social preference models are likely to capture mechanisms that permit people to play optimally in an evolutionary context. Once this context is taken into account, human behaviour no longer appears irrational.

The study of the one-shot anonymous ultimatum game (UG) has helped researchers to understand what influence social preferences^1^ have on human choice. In this game, two players—a *proposer* and a *responder*—are tasked with an economic decision^2^. First, the *proposer* suggests how to split a resource. This resource, provided by a third party, is set to 1.0. The offer defines the fraction that the proposer is willing to give to the responder. If the offer meets the responder’s demands (is accepted), the resource will be split accordingly. If the offer is rejected, neither player will receive anything. Our focus is on the standard version of the UG: a one-shot game between anonymous parties. A player’s strategy is defined as a vector (*offer*, *demand*). Note that these values are not probabilities but fractions within the range of [0, 1]. Therefore, strategies play deterministically, and the strategy space is theoretically infinite. The rational and most optimal strategy in this game differs markedly from the strategy observed in humans. This disparity has been explained by showing that humans take other elements, such as the social context, into account when making decisions. Here, we show that allowing players to discern between opponents that are kin and opponents that are not kin leads to the evolution of strategies that resemble those observed in humans—even if kinship detection is imperfect.

The UG has been extensively studied computationally in contexts such as spatial situations^3^,^4^, in games played on networks^5^,^6^, in closely related games^7^,^8^, and in the absence of complete information^9^. As we were interested in the *evolution* of strategies, we used a computational evolutionary model^10^,^11^,^12^ that defined a population of strategies as a set of points within the strategy space. We randomly selected a set of strategies to form the population of the first generation. These selected strategies all played against each other, and not against every possible strategy—much as an organism does not interact with all other possible organisms, but only with those alive at the time. The next generation was formed from those strategies that earned a higher payoff playing the UG. Specifically, the probability of an organism having offspring was defined by the payoff it received divided by the total payoff the entire population received (see Methods and Supplementary Information for a full description of the model). However, mutations could move the points defining the offspring’s strategy around in this space, providing a new set of opponents. How this set of points moved through this space over time approximated how evolution would act on organisms whose existence depended solely on playing the UG.

Our goal is to appropriately model the evolution of strategies playing the UG and to compare them with human behaviour. Unfortunately, the strategy vectors of humans (*offer*, *demand*) cannot be derived instantly from observing them play. Instead, offer distributions are reported in terms of empirical observations, and demands are presented as acceptance rates, conditioned on the offer. Thus, we first translate observations of human behavior into a strategy vector that can be directly compared with our findings obtained from computationally evolving strategies to play the UG. We then endowed these strategies with the ability to detect kinship relations. By comparing the trajectories of strategies playing the UG with and without the ability to detect kin, we were able to study the effect of kinship recognition on the optimality of evolved strategies.

From the point of view of economic rationality, however, how a set of strategies moves through space is not as important as how individuals optimize their own payoff. Specifically, the success of a strategy depends on its ability to optimize the offer in such a way that the smallest acceptable fraction is handed over. At the same time, demand has to be optimized such that the responder receives as much as possible and does not become too renunciative. The Nash equilibrium^13^,^14^ for the UG is to offer the lowest possible amount that is not zero, while at the same time accepting any non-zero offer (*offer* ~ 0.0, *demand* ~ 0.0). This can be directly derived when considering the subgame perfect equilibrium^15^,^16^: The responder has to choose between the present offer or nothing. Therefore, a responder with purely self-regarding preferences (i.e., maximization of self-interest) will accept any offer that is larger than zero, because anything is better than nothing. The proposer knows this, and can thus maximize her payoff by making the smallest possible non-zero offer. Offers optimized according this rationale should approach zero.

However, the strategy vector revealed in human play deviates^2^,^17^,^18^,^19^,^20^,^21^,^22^,^23^ from the rational Nash equilibrium (see the initial study by Güth *et al.*^2^; for a meta-analysis, see Spiegel *et al.*^24^) on three dimensions. First, the human strategy is more *generous*^25^; that is, proposers frequently offer more than the lowest possible amount above zero. The second dimension concerns *fairness*. Fairness is defined as making an offer that one would oneself be willing to accept, and requires the demand to be lower than one’s own offer (*demand* < *offer*)^10^. Thus defined, fair strategies offer more than they themselves demand. Not only do they play well against themselves, but they also do not expect something that they are not willing to give^11^,^12^. Note that fairness differs from inequity aversion^26^. Individuals can have advantageous or disadvantageous inequity aversion which, in the extreme case, causes them either to split the resource equally or to expect equal shares, which in the UG would be similar to a strategy of *offer* = 0.5 and *demand* = 0.5. Fairness also differs from altruism, which would require an altruistic individual to perform actions that improve the fitness of another individual at its own expense. In fact, humans do not necessarily demand less than they offer themselves, and thus do not play *fairly* as defined by Rand *et al.*^10^. Third and finally, it seems reasonable to assume that offers above 0.5 will nearly always be accepted^27^, across cultural differences and with few exceptions^28^,^29^; thus, humans are *demanding (demand* > 0.5), in that they accept all offers above 0.5 and have a diminishing acceptance rate for offers below 0.5.

## Results

In the UG players either make an offer or accept/reject an offer made by the other player, and it is these values (*offer* and *demand*) that we evolved in the computational model. In order to compare the results of the model with human play, we have to express human play as those two values. First, we asked how demanding humans really are. When observing humans, one can gauge a probability distribution for the offer (*p*_*offer*_(*offer*)), whereas the demand is typically reported as an acceptance rate dependent on the offer (*P*_*acceptance*_(*offer*)). Inferring demand from aggregated acceptance rates is problematic, however, because the acceptance rate is a function of the offers in question and thus not an independent measure.

For illustration, imagine an UG in which proposers make only unusually high offers between 0.75 and 0.85. Using the conventional approach of averaging over measured acceptance rates, which here would be 1.0 in each case, would result in a high demand (~0.8). Now assume an UG in which the same two responders encounter two proposers who make extremely low offers, say, 0.2 and 0.4. If we averaged across acceptance rates, the same two responders would suddenly have a much lower demand. As an alternative approach, we will show how to measure the demand of a responder *independently* of the offer distribution. When demand was measured in this way, we found that humans do not play fairly. Specifically, the demand was higher than the player’s own offer, violating the aforementioned definition of fairness^10^.

**Figure 1:**
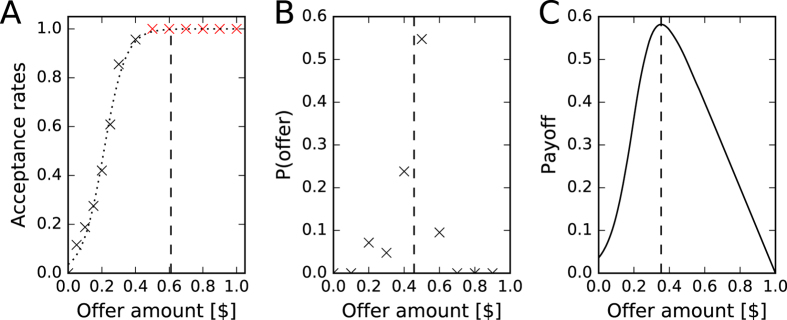
(**A**) Observed acceptance rates for offers between 0 and 0.5 in black, together with acceptance rates of 1.0 for offers above 0.5 in red (data from Harlé *et al.*^30^); the data were fitted to a logistic equation (Eq. 2, dotted line). The demand is indicated by the dashed line at 0.61. It indicates the threshold at which half of all possible offers, described by the fitted logistic function, were accepted. (**B**) Offer distribution from Forsythe *et al.*^31^ with a mean of 0.456 (dotted line), indicating that humans offered on average about 0.45. (**C**) Dependence of payoff (y axis) given a particular *offer* (x axis) defined by Equation 3, using the demand distribution *f* (*offer*) (shown in A). This curve shows that an offer of around 0.38 (indicated by the dashed line) had the highest expected return given the demand distribution estimated in A.

In the present analysis, we quantified demand by using data from an actual study of the UG but *not* conditioning it on offers^30^ (the findings in the present control group were consistent with those of many other experiments^2^,^17^,^18^,^19^,^20^,^21^,^22^,^23^,^24^). Specifically, we define the demand as the threshold that accepts half of all offers and rejects the other half. We fitted the data from the study together with an acceptance rate of 1.0 for offers above 0.5, because those offers are nearly always accepted (see above), to the logistic equation:


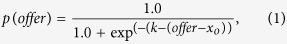


resulting in an optimal fit for *k* = 15.52 (the slope of the logistic function) and *X*_0_ = 0.21 (the x-value of the sigmoid’s midpoint). (The dotted line *f* (*offer*) in Fig. 1A shows the fit.) The *demand* was then found by solving the following equation for *offer*:


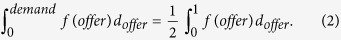


In contrast to previous studies quantifying the demand given specific offers (usually below 0.5), we found that the demand was well above 0.5 (see Fig. 1A).

Having described the demand of humans as a single threshold, we next asked whether this threshold was optimal given the measured offer distribution. Offers are normally distributed around a mean somewhere between 0.3 and 0.5 (depending on the study^10^,^31^; the data shown in Fig. 1B are from Forsythe *et al.*^31^). Assuming the demand distribution we inferred above, which included offers above 0.5, we assessed which offer would return the highest payoff. When *f* (*offer*) defines the likelihood that an offer will be rejected, the payoff can be quantified as:


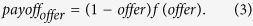


We found the payoff to be maximal at an offer of ≈0.38 (see Fig. 1C). Thus, the mean offer made by humans turned out to be optimal for the demand we measured. Therefore, we suggest that the strategy humans play is roughly (0.4, 0.6). This strategy is far from the rational Nash equilibrium (*offer* > 0), inasmuch as it is *generous* (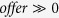) as well as *demanding (demand* > 0.5) and *unfair (offer* < *demand*). However, if played against an opponent who meets the demands and accepts the offer, this strategy provides an advantage. Specifically, it offers less than half of the resource, demands more than half of it, and results in a payoff that is larger than that of the opponent. Interestingly, this finding also follows our intuition about what it takes to win this game: As the proposer, one should hand over less than one retains (*offer* < 0.5); as the responder, one should receive more than the proposer retains (*demand* > 0.5).

Of course, the strategy proposed here is dependent on the context of the game it is played in ref. 27. For example, the exact probabilities can depend on gender^20^ as well as on hormonal levels^23^,^32^. However, the behaviour observed in these cases deviated even more from the rational Nash equilibrium than the proposed strategy. Our analysis was conducted to approximate the strategy humans play, and taking other contextual effects into account would not change our conclusions.

### Generous strategies are more adaptive

What drives humans to deviate from the rational Nash equilibrium? Social preference models explain the deviation by assuming that humans are motivated by considerations other than purely self-regarding ones, such as inequity aversion^33^,^34^, reciprocity^35^,^36^, fairness^10^,^17^,^18^,^34^,^37^, altruism, and punishment^38^,^39^,^40^,^41^,^42^,^43^. Alternatively, it has been argued that few real-world games are truly anonymous and one-shot^44^,^45^,^46^,^47^, or that during evolution games are repeated over generations, even though individuals might be confronted with “once in a lifetime” decisions (for details, see Binmore 2012^48^). Repeated encounters allow players to deploy different strategies, so that, for example, players stop cooperating with notorious defectors. Similarly, the green-beard effect^49^,^50^ could affect the UG. The green-beard effect describes a situation in which a particular trait (the green beard) is recognized by other organisms, and permits conclusions to be drawn about organisms with that trait. In the UG, players recognized in this way might elicit different responses—for example, a particularly demanding strategy might receive a higher offer. The notion of repeated games is independent from kin detection, because the latter can also operate in one-shot games and independently of a green-beard effect. In sum, all these accounts help to explain why humans do not play the rational Nash equilibrium, and why their behaviour is inconsistent with the canonical economic model that considers humans to be rational and self-regarding.

Complementing these accounts, we propose that humans, being social animals, evolved behaviour that is highly influenced by kin selection^49^,^51^. Specifically, we suggest that humans assume that the anonymous opponent is not likely to be kin-related. Consequently, they play a strategy in the UG that is optimal against non-kin opponents. Humans and their evolutionary ancestors were capable of detecting kin, and there is ample evidence for an evolved capacity to detect kinship relations in humans and animals^52^. Next, we will show that strategies that cannot discern between kin and non-kin evolve such that they play the rational Nash equilibrium. In contrast, strategies that are empowered to play differently against kin and non-kin, based on their ability to detect kinship relations^49^,^51^, evolve to be *generous* (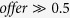), *unfair (offer* < *demand*), and *demanding (demand* > 0.5).

### Kin detection, kin selection

**Figure 2:**
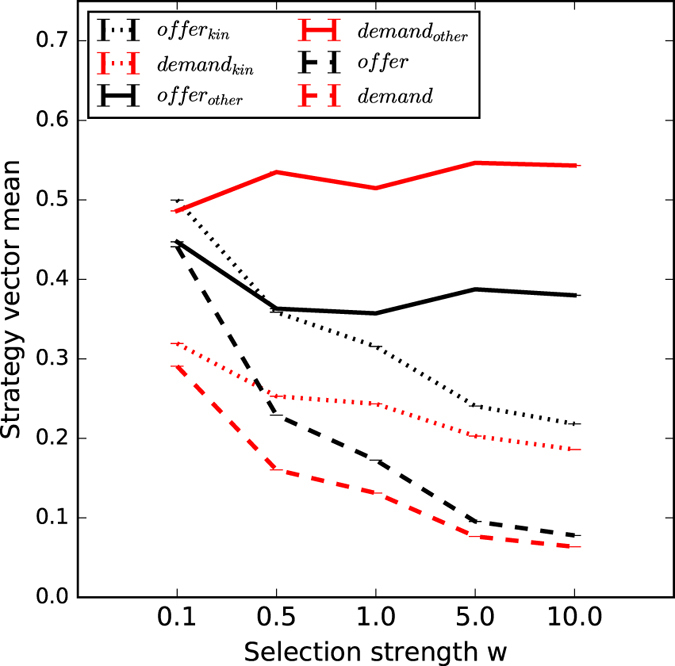
Mean evolved *offer* (black) and *demand* (red) at different selection strength (*w*, x axis), for two conditions. Dashed lines show strategies evolved without kin detection and resemble the findings from Rand *et al.*^10^. For strategies permitted to evolve kinship detection, dotted lines represent *offer*_*kin*_ and *demand*_*kin*_ when playing against kin, whereas full lines represent *offer*_*other*_ and *demand*_*other*_ when playing against others detected as non-kin. Error bars indicate standard error. Due to the large number of data points, error became vanishingly small.

In evolving strategies to play the anonymous one-shot UG without the ability to detect kinship, we confirmed the results from Rand *et al.*^10^ (see dashed lines in Fig. 2): Agents evolved the behaviour defined by the rational Nash equilibrium. Furthermore, increasing the strength of selection increased the propensity of agents to play the thus defined rational strategy. Consequently, the evolution of strategies unable to detect kinship cannot explain why humans play in a way that is generous, unfair, and demanding^10^.

In the next step, we introduced the ability to detect kinship. In the computational evolutionary model, each agent received a *tag* ∈ [0, 1] and a threshold to recognize kin *t* ∈ [0, 1]^53^,^54^. Let us imagine two strategies, *A* and *B*. Strategy *A* will recognize strategy *B* as kin if the difference between the two tags is smaller than the recognition threshold of *A* (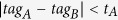). Note that strategy *B* could come to a different conclusion than *A*. For illustration, assume *tag*_*A*_ = 0.5 and *tag*_*B*_ = 0.7, so that the difference between their tags is 0.2. If the threshold of *A* is *t*_*a*_ = 0.3, *A* will recognize *B* as kin. However, *B*’s threshold might be stricter (*t*_*B*_ = 0.1), meaning that *B* does not recognize *A* as kin. To permit strategies to play conditionally on the kin tag, we had strategies use one pair of values (*offer*_*kin*_, *demand*_*other*_) when recognizing the other party as kin and another pair of values (*offer*_*other*_, *demand*_*other*_) when recognizing the other party as non-kin. Note that if a strategy experienced a mutation of the *tag* in such a way that it became close enough to the *tag* of another strategy, it would recognize it as kin. In this way, non-kin strategies can be misinterpreted as kin.

When evolving strategies that are able to detect kin, we have to distinguish between strategies that classify the other party as kin and strategies that classify the other party as non-kin. If the evolved strategy decides that the other party is kin, its behaviour will be similar to that predicted by the rational Nash equilibrium (see dotted lines in Fig. 2). However, if the evolved strategy decides that the other party is non-kin, a much more generous strategy with a high demand will evolve (see solid lines in Fig. 2). Based on the latter decision, the evolved strategy will produce behaviour that is very similar to that observed in humans (see also Fig. 1), namely the play of this evolved strategy (0.4, 0.6) will be *generous* (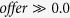), *unfair (offer* < *demand*), and *demanding (demand* > 0.5).

**Figure 3:**
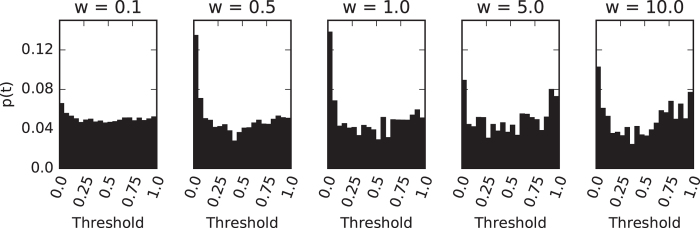
Distribution of evolved kin recognition thresholds. The histograms show the distribution of the kin recognition threshold recovered from the line of decent for different selection strength *w* ranging from 0.01 (left) to 10.0 (right) as annotated above. Except for the weakest selection strength we find a preference for low thresholds around 0.0.

Although the *tag* did not converge on a particular value (data not shown), we found that for higher strength of selection (*w* > 0.1) the threshold (*t*) evolved to be small, indicating that agents were indeed selected to detect kin (see Fig. 3). The noise on the threshold was uniform and can be explained by random mutations^55^, indicating that the threshold wasn’t under strong. It seems likely that successful strategies gave rise to offspring with identical tags which in turn did not require low thresholds. Once the tag mutates in an individual, the threshold needs to be low again in order to discriminate between the new mutants. This back and forth might explain the limited selection strength.

The ability to discern between kin and non-kin permited players to deploy two different strategies. This has profound implications for the evolutionary stability of strategies^56^. Simply put, a strategy has to fare better against itself (its own kin) than other strategies that play against it to become evolutionarily stable (defined by Smith 1982^56^, as the relation between payoffs *E* that strategies receive: *E*(*self*, *self*) > *E*(*other*, *self*)). By detecting kin, a strategy can maximize the payoff it receives against itself (*E*(*self*, *self*)), independently of what other strategies receive when playing it (*E*(*other*, *self*)). Therefore, evolved strategies in the UG maximized their payoff when playing against themselves by playing what the rational Nash equilibrium dictates. This behaviour is also robust against invasion by others who are misclassified as kin. At the same time, the branch of the strategy that played against non-kin had the ability to maximize its own payoff while minimizing the gains of others. This was achieved by offering less than 0.5 and demanding more than 0.5 (see the Supplementary Information for details). Increasing the minimal recognition threshold gradually compromised the ability to detect kin. Consequently, strategies cease to be generous, fair, and demanding. Ultimately, they returned to the strategy defined by the rational Nash equilibrium.

## Discussion

Our results were obtained by computationally evolving strategies to play the UG conditionally on a kin tag and comparing the results with observations of human play. The model used a well-mixed population, and reduced behaviour in the UG to an offer and demand threshold. Interesting opportunities to expand the model in the future would be to include more complex strategies, iterated play, and spatial structures, but we do not expect any of these additions to fundamentally change our results. The model also assumed that humans use a single demand threshold, which we derived from experimental data for offers below 0.5, and we assumed all offers above 0.5 to be accepted. Future experiments that measure the demand independently from the actual offer (e.g., with the help of the strategy method^57^) might be able to model human behaviour more accurately, and would thus allow a more precise comparison between computationally evolved strategies and human behaviours.

We found that humans play the very same strategy in the UG that computationally evolved strategies played against non-kin others. We therefore suggest that humans, thrown into the UG, assume the anonymous other to be non-kin. In addition, we found that evolved strategies that assumed the other to be kin played a strategy similar to that described by the rational Nash equilibrium. Both of these findings are consistent with findings about the impact of genetic relatedness and with the offer and demand distribution^22^ in the UG. For instance, genetically highly related villagers in Dominica^58^ have been found to demand much less (0.152) than genetically unrelated Amazon Mechanical Turk workers^10^ (0.25 < *offer* < 0.5). There may also be other reasons for humans in small communities to play less demanding; these possible reasons deserve to be explored in the future.

Although these results are quite astonishing and offer an explanation for human behaviour in the UG, the computational model is abstract. Of course, humans have not evolved to play the UG, and we cannot rule out other influences on human behaviour in this game. However, the computational model is general enough to be applicable to all organisms that have the ability to detect kin. Our analysis suggests that kin detection not only benefits kin, but also enables the evolution of generosity towards others who are not kin.

Our results also offer a new interpretation for social preference models. The robust finding that humans routinely deviate from the rational Nash equilibrium in the UG has led some to conclude that human behaviour is not fully rational in the terms of the canonical economic model. Social preference models have been proposed to explain the lack of full rationality. Here, however, we find that strategies that can distinguish between kin and non-kin evolve their *offer* and *demand* to be similar to those found in humans. This suggests that the human strategy is not necessarily irrational; rather, that is it the rational strategy if kinship relations are considered. Thus, social preference models might capture the psychology that enables humans to play the evolutionarily optimal strategy.

## Methods

The one-off UG was implemented as described^10^ before. Kinship detection was also added as described^53^,^54^ before. Two-hundred independent evolutionary runs were performed per condition, using independent random number seeds for each replicate. The population was started with random genomes. Population size was 100 and the mutation rate was 0.01 for *offer* and *demand* as well as for the tag and the recognition threshold. The mutation rate was implemented as a per site mutation rate, so that each value of the strategy vector could mutate independently. If a mutation occurred, a new value was drawn from a uniform random distribution [0, 1]. At the end of each run, the line of descent (LOD) was reconstructed^59^ and the results were averaged over the LOD. Using the population mean instead of the LOD made no significant difference to the results (data not shown). The code and the analysis software can be downloaded from https://github.com/ahnt/UltimatumGame.

## Additional Information

**How to cite this article**: Hintze, A. and Hertwig, R. The Evolution of Generosity in the Ultimatum Game. *Sci. Rep.* **6**, 34102; doi: 10.1038/srep34102 (2016).

## Supplementary Material

Supplementary Information
