# Supplementary material for: The Evolution of Generosity in the Ultimatum Game
Source: Sci Rep. 2016 Sep 28;6:34102. doi: 10.1038/srep34102 (PMC5039727; doi:10.1038/srep34102)
Supplement: Supplementary Information [file srep34102-s1.pdf]

The Evolution of Generosity in the Ultimatum Game

Arend Hintze[1,2,3] & Ralph Hertwig[4]

BEACON Center for the Study of Evolution in Action,

Department of Computer Science and Engineering,

Department of Integrative Biology,
Michigan State University, East Lansing, USA

Center for Adaptive Rationality,
Max Planck Institute for Human Development, Berlin, Germany

Supplementary Information

In order to understand why the detection of kinship allows strategies to become generous, we have to understand under which conditions strategies become evolutionarily successful. Let us consider two strategies i and j playing the one-shot anonymous ultimatum game. In this game, the first player has to make an offer on how to split a resource. This resource is set to 1.0. The offer is the fraction that the first player (proposer) offers to the receiving second player (responder). If the offer meets the responder's demands, the resource is split accordingly. If the offer is rejected, neither player receives anything. The game is played so that each player interacts with every other player, once as proposer and once as responder. The player's strategy is defined by a pair of values (p, q), where p is the fraction offered, and q is the demand to be met.

The success of a strategy depends on its ability to optimize the offer (p) in such a way that the smallest acceptable fraction is handed over. At the same time, the demand (q) has to be optimized such that the responder receives as much as possible and does not become too renunciative. The Nash Equilibrium (NE) for this game is $p \approx \epsilon > 0.0$ and $q \approx \epsilon > 0.0$ (where ϵ is the smallest value one can offer that is not 0.0; in the following, we refer to this as $p = 0.0$ or $q = 0.0$) and can be derived directly from considerations about the evolutionarily stable strategy (ESS) [1].

The evolutionary stability of a strategy i or j depends on the payoffs players receive playing each other $E(i, j)$ and $E(j, i)$, as well as playing against themselves $E(i, i)$ and $E(j, j)$ (see Table S1).

	i	j
i	$E(i,i)$	$E(i,j)$
j	$E(j,i)$	$E(j,j)$

Table S1: Payoff E defined by interactions of strategy i and j

The strategy i can become an ESS [1] if:

$$E(i,i) > E(j,i) \tag{0.1}$$

or if:

$$E(i,i) = E(j,i) \tag{0.2}$$

and

$$E(i,j) > E(j,j) \tag{0.3}$$

The above statement can be rephrased as: A strategy is an ESS if the payoff it receives playing itself $E(i, i)$ is larger than the payoff any other strategy receives playing it, $E(i,i) > E(j,i)$. Thus, in order to find ESSs, one needs to look at the diagonal, and every strategy whose payoff on the diagonal is larger than any payoff in the same column is an ESS (see Table S2).

The evolutionary stability of strategy i does not exclude other strategies $(j, k, l, ...)$ from also being evolutionarily stable. To simplify the payoff matrix E, one can subtract any constant in each column without changing the evolutionary stability of the strategies. Therefore, we arrange the matrix in such a way that we have only 0 on the diagonal. The outcome of a game is now defined only by the sign of each value off the diagonal[2]. Ideally, for strategy i to become an ESS, it should maximize the payoff if receives against j and minimize the payoff strategy j receives playing i (see Table S2). Maximizing the payoff a strategy receives against itself increases its value on the diagonal, which increases its chance of being the highest value in the column. Conversely, increasing the payoff a strategy receives against another strategy reduces the likelihood that the opponent strategy has a higher payoff playing against itself then playing against the first strategy.

In order to optimize the payoff while remaining an ESS (and preferably the only strategy to be an ESS), p and q have to change. Table S3 shows in green that for strategy i to receive more than j, q should be larger than 0.5 and p should be smaller than 0.5. At the same time, strategy i must

	i	j
i		maximize
j	minimize	

Table S2: Payoff E, and what strategy i has to change in order to become the only strategy to be an ESS

play against itself, and fairness dictates that $p > q$ [3]. Thus, the optimal strategy to play this game is $p = 0.0$ and $q = 0.0$ (while $p > q$), which is the ESS and the NE. However, this case considers only strategies that play unconditionally regardless of the opponent. If a strategy could lift the fairness constraints ($p > q$) by playing differently against itself than against other strategies, q could become larger, as Table S3 suggests. The ability to detect kin would allow this to happen.

	i	j
i	$p > q$	$q > 0.5$
j	$p < 0.5$	

Table S3: Necessary condition for strategy i to become an ESS in red, and possible optimizations for strategy i to become the only strategy to be an ESS in green

To test this hypothesis, we introduce the ability for strategies to detect kinship. Each agent receives a *tag* and a kin recognition threshold (t), where each value will be held between 0.0 and 1.0[4, 5]. A strategy i recognizes another strategy j as kin if $|tag_i - tag_j| < t_i$. Note that strategy j could come to a different conclusion than j. For illustration, let $tag_i = 0.5$ and $tag_j = 0.7$; $t_i = 0.3$ and $t_j = 0.1$. In this situation, i will recognize j to be kin, because $|0.5 - 0.7| < t_i$. Strategy j, on the other hand, will not recognize strategy i to be kin because $|0.5 - 0.7| > t_j$. To permit strategies to play conditionally on the kin tag, we had strategies use one pair of values (p_{kin}, q_{kin}) when recognizing an opponent as kin and another pair of values (p_{other}, q_{other}) when recognizing an opponent as non-kin. Strategies still have to obey the basic rules in order to become an ESS (see Eq. 0.1 and 0.3), and strategy i can still try to prevent other strategies from being ESSs by maximizing $E(i, j)$ while minimizing $E(j, i)$ (see Table S3).

But now that an agent can potentially play differently against agents who are kin, we obtain a different payoff table (See Table S4).

	i	j
i	$\begin{cases} 1 & \text{if } p_{kin} > q_{kin} \\ 0 & \text{if } p_{kin} < q_{kin} \end{cases}$	$\begin{cases} 1 - p_{other} & \text{if } p_{other} > q_j \\ 0 & \text{if } p_{other} < q_j \end{cases} + \begin{cases} p_j & \text{if } p_j > q_{other} \\ 0 & \text{if } p_j < q_{other} \end{cases}$
j	$\begin{cases} p_j & \text{if } p_j > q_{other} \\ 0 & \text{if } p_j < q_{other} \end{cases} + \begin{cases} p_{other} & \text{if } p_{other} > q_j \\ 0 & \text{if } p_{other} < q_j \end{cases}$	$\begin{cases} 1 & \text{if } p_j > q_j \\ 0 & \text{if } p_j < q_j \end{cases}$

Table S4: Payoff table for strategy i and j assuming that strategy i can detect kin. Strategy i is described as $(p_{kin}, q_{kin}, p_{other}, q_{other})$, while strategy j is simplified to (p_j, q_j)

This new payoff matrix allows strategies to be fair, because $p_{kin} > q_{kin}$, while at the same time having a higher demand $q_{other} > 0.5$ and a lower offer $p_{other} < 0.5$.

This explains how introducing the ability to detect kinship relations can result in a strategy becoming generous toward non-kin players while still remaining an ESS.

Limiting Kin Detection

As a control to test the effect of kin detection, we manipulated the minimal selection threshold. Instead of having the lowest threshold set to 0.0, we systematically increased the threshold in 0.1 intervals and repeated the evolutionary experiment. The results (See Figure S1) confirm the intuition that when removing the effect of kin selection, the evolved strategies playing against kin become more and more similar to the Nash Equilibrium, while the part of the strategy that is used against non-kin starts to drift until it becomes completely neutral at very high threshold levels, because at high minimal thresholds no opponent is recognized as kin. A value not under selection will drift randomly, and since we sample from a uniform random distribution ($[0.0, 1.0]$) we can identify a completely neutral value as having a variance of $\frac{1}{12}$, since that is the variance of a uniform random distribution. This also confirms that the effects on the $offer_{other}$ and $demand_{other}$ are not due to drift but to actual selection pressures.

Computational Model Implementation

The computational model is written in C++; the code can be obtained here: (A link to the code will be provided upon acceptance of the manuscript). The code is structured into three sections: setup, evolution, data output. While specific experiments might change certain parameters, here we describe the default behaviour. At program start a population of agents is created, where each agent receives a genome vector that contains four values drawn from a uniform random distribution [0,1]. The first two values describe the *offer* and *demand* used when the opponent is recognized as kin; the other two values specify the *offer* and *demand* used when the opponent is not recognized as kin. Additionally, the tag is drawn from a uniform random distribution [0,1], and the recognition threshold is drawn from a distribution [limit,1]. The limit is used to test limits in kin detection as described above. At each generation (replacing one agent at a time) a random agent is removed, and needs to be replaced by another one proportional to fitness. Thus, we compute the likelihood for an agent to propagate offspring into the next generation as the agents score divided by the sum of all scores. After that we randomly select an agents and draw a random number ($[0.0, 1.0]$) and test if this random number is smaller than the agentâs relative score. If so, this agent is used to replace the one that got removed; if not we continue to randomly draw agents. This mechanism ensures that agents are being replaced proportional to their score. After 5,000,000 of these generations, a random agent is picked, and we record all values of all agents preceding the agent picked, back along its list of ancestors to an individual in the first generation. These data are then saved to a file and constitute one experiment. Each experiment uses a new and independent random seed.

Starting Condition Independence

At program start, agents are initialized with random values. To exclude the possibility that the starting point affected the outcome of the experiment, we tried various starting conditions, and found that the evolutionary trajectories converged on the same point, regardless of start conditions. Because each organism is initialized with six values, we started independent experiments with any combination of these six values being either 0.0 or 1.0, resulting in 64 possible start conditions. All other experimental conditions were identical to the evolutionary experiment described for Figure 2 (main text) for $w = 10.0$ and allowing for kin detection to evolve. Each experiment was repeated 100 times; lines of descent were constructed, and data points after 2.5 million updates averaged to find the convergence point. We found that, within the expected slight variation, the starting point had no effect on the point of convergence (see Figure SS2). At the same time, we again found that, independently of the start condition, p_{self} and q_{self} evolved to be fair ($p > q$) as indicated by the dashed line in Figure S2.

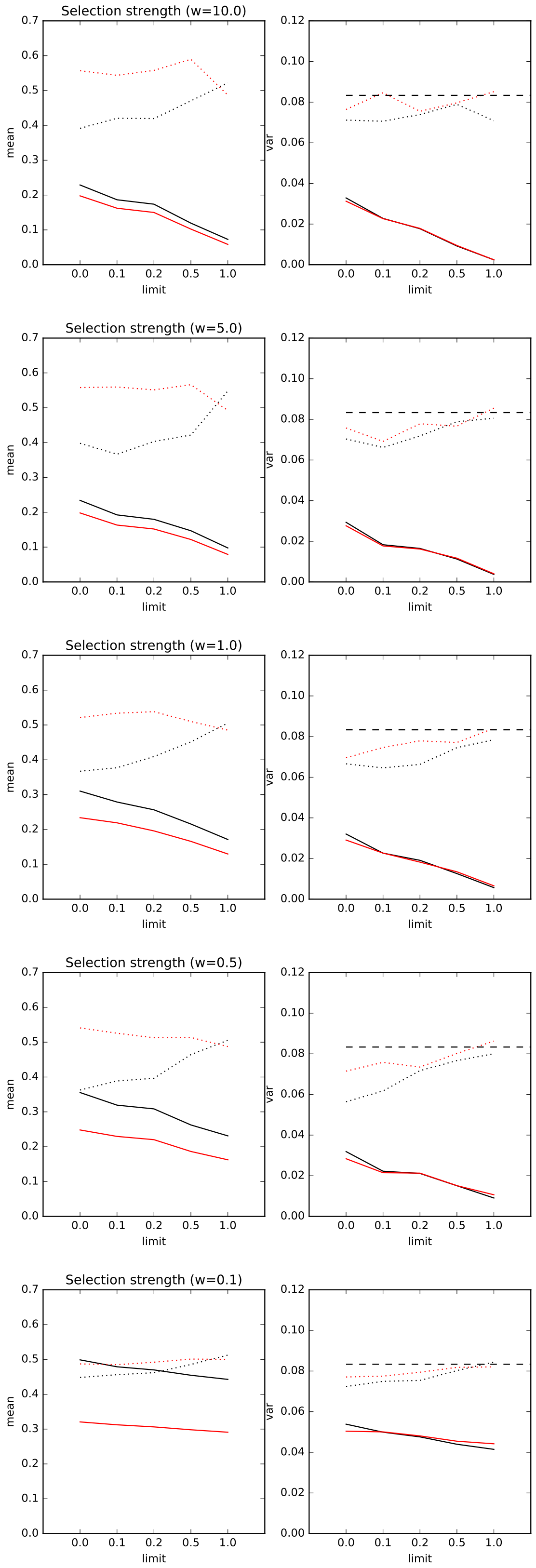

Figure S1: Mean evolved *offer* (black) and *demand* (red) at different selection strength (w), and different minimal kin detection thresholds (x axes): dotted lines represent $offer_{kin}$ and $demand_{kin}$ for strategies used when playing against kin, whereas full lines represent $offer_{other}$ and $demand_{other}$ for the same strategy playing against opponents recognized as non-kin.

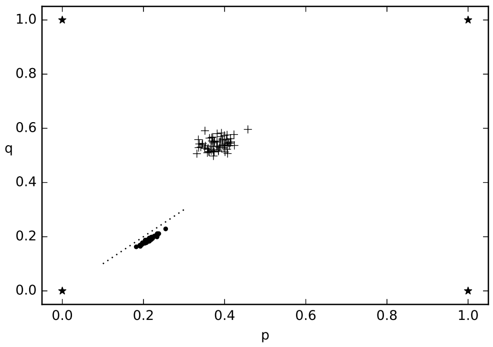

Figure S2: Start condition independence. Start conditions are indicated by a star. The p_{self} and q_{self} from all 64 start conditions tested are shown as dots; their corresponding p_{other} and q_{other} by plus signs. The dotted line indicates $p = q$ and marks the threshold for strategies to be considered fair.

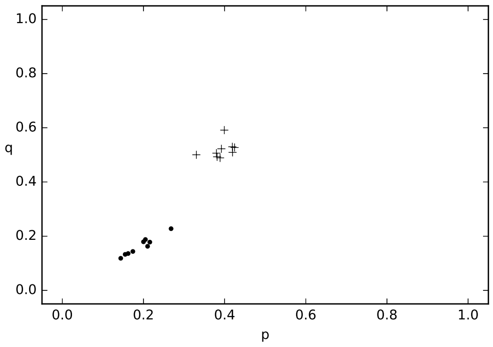

Figure S3: The p_{self} and q_{self} from all nine combinations for my (0.001, 0.01, and 0.02) as well as population sizes N (100, 500, and 1000) are shown as dots; their corresponding p_{other} and q_{other} as plus signs.

Population Size and Mutation Rate

To rule out the possibility that mutation rate my (0.01) or population size (100) affected the outcome of the experiment, we explored two more mutation rates (0.001 and 0.02) and other population sizes (500 and 1000) in combination with each other. For each of the nine parameter combinations, 100 replicate runs were performed with selection strength $W = 10.0$. As in the starting condition experiment, the averages of p_{self} and q_{self} after 2.5 million updates were computed, as well as their respective values of p_{other} and q_{other}. As shown in Figure S3, there were no noticeable differences. Implementing even larger or even smaller mutation rates and population sizes than the ones implemented would be increasingly unrealistic.

References

[1] Smith, J. M. *Evolution and the Theory of Games* (Cambridge Univ. Press, 1982).

[2] Zeeman, E. C. Population dynamics from game theory. In *Global Theory of Dynamical Systems*, 471–497 (Springer, 1980).

[3] Rand, D. G., Tarnita, C. E., Ohtsuki, H. & Nowak, M. A. Evolution of fairness in the one-shot anonymous ultimatum game. *Proc. Natl. Acad. Sci. USA* **110**, 2581–2586 (2013).

[4] Riolo, R., Cohen, M. & Axelrod, R. Evolution of cooperation without reciprocity. *Nature* **414**, 441–443 (2001).

[5] Traulsen, A. & Schuster, H. G. Minimal model for tag-based cooperation. *Phys. Rev. E. Stat. Nonlin. Soft Matter Phys.* **68**, 046129 (2003).
